# Atrial fibrillation detected after stroke: Recent advances and future directions

**DOI:** 10.1177/17474930261465951

**Published:** 2026-07-05

**Authors:** Lussiana Folleco-Insuasty, Luis Fernando Roa-Wandurraga, Federico Liberman, Diana Ayan, Rodrigo Bagur, Luciano A Sposato

**Affiliations:** 1 Neurology Unit, Hospital Universitario San Ignacio, Pontificia Universidad Javeriana, Bogotá, Colombia; 2 Department of Medicine, Division of Cardiology, Schulich School of Medicine and Dentistry, Western University, London, ON, Canada; 3 Heart and Brain Laboratory, Schulich School of Medicine and Dentistry, Western University, London, ON, Canada; 4 Department of Clinical Neurological Sciences, Schulich School of Medicine and Dentistry, Western University, London, ON, Canada; 5 Robarts Research Institute, Schulich School of Medicine and Dentistry, Western University, London, ON, Canada; 6 London Health Sciences Centre Research Institute, London, ON, Canada; 7 Department of Clinical Neurological Sciences, London Health Sciences Centre, University Hospital, Western University, London, ON, Canada

**Keywords:** Atrial fibrillation, AFDAS, ischemic stroke, transient ischemic attack, cardiac monitoring, implantable cardiac monitor, anticoagulation

## Abstract

Prolonged cardiac monitoring has made atrial fibrillation (AF) an increasingly common diagnosis after ischemic stroke. Applied to all patients with ischemic stroke, prolonged cardiac monitoring would result in over a million new AF diagnoses per year worldwide. Almost 10 years after the conceptual inception of AF detected after stroke (AFDAS), several patterns are now consistent across studies. AFDAS is a distinct, mechanistically heterogeneous entity that differs from AF known before stroke in its risk-factor profile, cardiac comorbidity, and its lower rate of ischemic stroke recurrence. Its detection is determined primarily by the monitoring method and increases with earlier and longer-duration monitoring. AF identified on a standard electrocardiogram behaves as a high-burden arrhythmia, resembling AF known before stroke, and should be distinguished from the low-burden AF detected by prolonged monitoring. Important questions remain unresolved. It is unproven that detecting more AF, or detecting it earlier, reduces recurrent ischemic stroke. The low-burden arrhythmia that monitoring increasingly identifies appears to carry a lower embolic risk than the high-burden AF for which anticoagulation was established. Whether patients with low-burden, monitor-detected AFDAS derive net benefit from anticoagulation, the contribution of neurogenic mechanisms, and the thresholds of AF burden that should guide treatment are undefined. Future trials should be powered for recurrent stroke as the primary endpoint.

The detection of atrial fibrillation (AF) after ischemic stroke has changed faster than almost any other element of secondary prevention.^1,2^ A decade ago, a normal admission electrocardiogram (ECG) and brief inpatient telemetry were considered an adequate rhythm assessment. Implantable cardiac monitors (ICMs) now record cardiac rhythm for years, and AF yield increases with each increment in monitoring duration and intensity.^3^ If prolonged monitoring were applied to every patient with ischemic stroke, it could result in over a million new AF diagnoses per year worldwide.^4^ The capacity to detect AF has therefore expanded considerably. The evidence that this expansion improves acute ischemic stroke patient outcomes is not robust enough to warrant universal anticoagulation.

This issue of the *International Journal of Stroke* assembles five studies that map the clinical pathway of AF detected after stroke (AFDAS), a concept we introduced almost a decade ago to describe AF with a clinical profile distinct from AF known before stroke.^5^ We use these studies to discuss their contribution to our understanding of AFDAS and highlight persistent knowledge gaps.

## AFDAS: what is known

AF is newly diagnosed in approximately one-quarter of patients with ischemic stroke or transient ischemic attack (TIA) who have no previously recognized arrhythmia.^2^ Mechanistically, AFDAS is a heterogeneous entity arising from a cardiogenic substrate and potentially driven by neurogenic mechanisms in some cases. Over the past decade, evidence has converged on the concept that AF first detected after a cerebrovascular event on continuous cardiac monitoring differs from AF known before the stroke (KAF).^1,2^ Relative to KAF, AFDAS is associated with a lower prevalence of vascular risk factors, less structural and atrial cardiomyopathy, and a lower arrhythmic burden.^1^ These differences carry prognostic weight. In a meta-analysis of 17 studies and 113,365 patients (33,026 with AFDAS and 80,339 with KAF), AFDAS was associated with a lower risk of recurrent ischemic stroke (risk ratio = 0.79, 95% confidence interval (CI) = 0.66–0.95) and lower mortality (risk ratio = 0.84, 95% CI = 0.74–0.95), with a similar risk of intracerebral hemorrhage.^6^ The recurrence risk of AFDAS is not uniform and varies with the method and timing of detection. AF identified on a 12-lead electrocardiogram (ECG-AF) after stroke carries a recurrent stroke risk approximately fivefold higher than AF detected only on 14-day monitoring (adjusted hazard ratio = 5.06, 95% CI = 1.13–22.7), consistent with a higher arrhythmic burden among ECG-detected cases.^7^ In another study, these differences were time varying: AFDAS carried a durably lower recurrence risk than KAF (adjusted hazard ratio = 0.22, 95% CI = 0.08–0.63), whereas the lower recurrence of ECG-AF was confined to the first year and converged with that of KAF thereafter (adjusted hazard ratio at the end of follow-up = 0.67, 95% CI = 0.36–1.26).^8^ Detection method is therefore a marker of AF burden, and AF found on a standard ECG resembles KAF more than monitor-detected AFDAS. As such, in line with the 2024 classification, ECG-AF is not AFDAS.^1^

## AFDAS and the neurogenic hypothesis

Ababneh et al.^9^ conducted a large systematic review and meta-analysis of the prevalence and predictors of AFDAS, including 91 observational cohorts of patients with ischemic stroke or TIA for prevalence and 54 for predictors. They classified predictors into demographic, cardiogenic, neurogenic, and laboratory domains. Demographic and clinical predictors included older age, female sex, hypertension, and chronic kidney disease. Cardiogenic predictors included left atrial enlargement, advanced interatrial block, heart failure, coronary artery disease, reduced ejection fraction, PR interval prolongation, QRS duration, and cardioembolic stroke subtype. Neurogenic predictors, including higher admission stroke severity on the NIHSS and insular cortex involvement, were interpreted as markers of stroke-related disruption of cardiac autonomic control. The strongest reported associations included admission NIHSS (odds ratio (OR) = 8.07, 95% CI = 3.10–20.99), cardioembolic stroke subtype (OR = 8.71, 95% CI = 2.38–31.82), advanced interatrial block (OR = 6.55, 95% CI = 1.62–26.55), left atrial enlargement (OR = 5.75, 95% CI = 1.50–22.12), and insular involvement (OR = 2.90, 95% CI = 1.58–5.31). Elevated NT-proBNP and BNP were also associated with AFDAS.

The included studies were observational and clinically heterogeneous, statistical heterogeneity was high for most predictors, and several associations rested on few cohorts. The classification of predictors as cardiogenic or neurogenic was based on presumed mechanisms described in the source studies. Detection method also varied across cohorts, influenced AF detection yields, and included AF identified on emergency-room or inpatient ECG. As such, a proportion of the pooled cases likely represent ECG-detected AF instead of true AFDAS, which under the 2024 definition requires continuous cardiac monitoring.^1^ In addition, some apparent AFDAS may have been, in fact, unrecognized AF present before the stroke but never diagnosed. The authors acknowledge that this may have overestimated newly diagnosed AF incidence and confounded the analysis. We would therefore encourage future studies to report ECG-detected AF separately and to apply the diagnostic criteria we discuss below, which reserve the term AFDAS for AF detected by continuous cardiac monitoring (e.g. >7 days).

**Figure 1.**
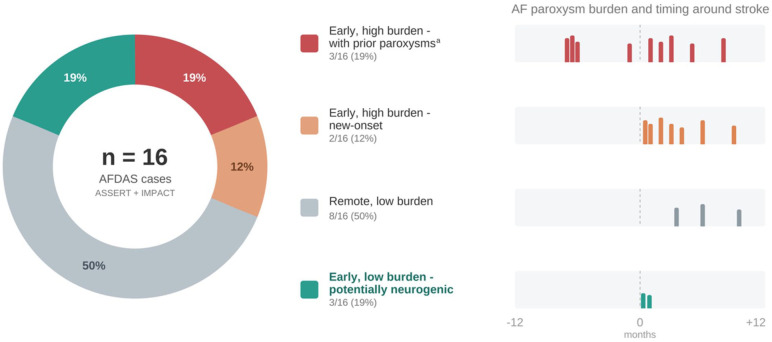
Clinical presentations of atrial fibrillation detected after stroke. The doughnut shows a schematic distribution of AFDAS presentations among 16 cases from the ASSERT (Asymptomatic Atrial Fibrillation and Stroke Evaluation in Pacemaker Patients and the Atrial Fibrillation Reduction Atrial Pacing Trial)^10,11^ and IMPACT (Randomized trial of atrial arrhythmia monitoring to guide anticoagulation in patients with implanted defibrillator and cardiac resynchronization devices)^12^ trials who had monitoring data before and after stroke, classified by arrhythmia burden and timing relative to the index event.^1^ For each presentation, the horizontal gray bar represents the period of cardiac rhythm observation, spanning 12 months before to 12 months after the stroke, and the vertical colored lines mark individual atrial fibrillation paroxysms, colored according to presentation type. The dashed vertical line indicates the time of the stroke. The early, low-burden pattern most consistent with a transient neurogenic trigger accounted for 3 of 16 cases (19%). AFDAS: atrial fibrillation detected after stroke; KAF: known atrial fibrillation. ^a^In the first group, AF was present before the stroke, so a proportion of apparent AFDAS may represent unrecognized known AF (KAF), unknown to clinicians and patients.

Two points deserve emphasis. First, an association with potentially neurogenic markers does not establish a neurogenic cause. Higher NIHSS scores and insular involvement also track stroke severity, a cardioembolic mechanism, and more intensive monitoring, each of which independently increases AF detection, and the largest associations in this synthesis were cardiogenic or severity markers, whereas the neurogenic associations were more modest. Consistent with this uncertainty, in a typology of 16 cases of AFDAS from the ASSERT and IMPACT trials, the early, low-burden pattern most compatible with a transient neurogenic trigger accounted for only 3 of 16 cases (19%) (Figure 1). This sample is small, and the B^2^AD-RISK AFDAS (Evolution of Burden of AF, Biomarkers, Left Atrial Characteristics, Demographics, and Risk Factors in AF Detected After Stroke) study is assessing these burden and timing patterns prospectively after stroke (NCT06589700).^1^ The true contribution of neurogenic mechanisms to AFDAS, therefore, remains uncertain.

As pointed out by the authors, the concurrent identification of atrial-substrate and stroke-related markers is consistent with a mechanistically heterogeneous syndrome, in which some cases reflect previously silent atrial cardiopathy and others may be promoted or unmasked by acute stroke. These findings support more personalized post-stroke rhythm monitoring and the development of risk models integrating cardiogenic, neurogenic, and biomarker data to guide anticoagulation decisions. For this purpose, future studies should consistently report AF burden following recent consensus statements, screening method, and duration of monitoring.^13^

## Timing of cardiac monitoring and yield of AF detection

Two studies in this issue address the timing of monitoring. In a target trial emulation of 333 patients with embolic stroke of undetermined source (ESUS) who received an ICM, D’Anna et al.^14^ found that implantation within 30 days of the index event was associated with higher AF detection at 30 days than delayed implantation (7.8% vs. 1.6%; OR = 4.49, 95% CI = 1.17–17.27), with consistent hazards at 90 and 120 days and a shorter time to diagnosis. Their companion systematic review and meta-analysis of 47 studies and 6918 patients reported a pooled ICM detection rate of 27.3% and a higher yield with early implantation before 31.5 days than with delayed implantation (30.0% vs. 23.7%), independent of monitoring duration.^15^ Together, these analyses provide consistent evidence that timing, and not duration alone, is associated with diagnostic yield.

One interpretation offered by D’Anna et al. is that the higher diagnostic yield with early ICM implantation may reflect the dynamic behavior of AF after stroke. AF is often intermittent and may be more readily captured when monitoring begins during the acute phase, when the pre-test probability of an AF-related mechanism is highest.

These findings should be interpreted cautiously. Both studies were observational. Although the target trial emulation and the meta-analysis attempted to reduce bias, neither design can eliminate residual confounding, selection bias, or confounding by indication. The authors note in particular that patients undergoing delayed implantation may constitute a selected subgroup, namely those who had not already experienced AF detection or recurrent stroke before the procedure, such that the lower yield in the delayed group may partly reflect the attrition of high-risk individuals, an effect distinct from the timing of implantation. Additional limitations include the small early-implantation group and the correspondingly wide confidence interval in the emulation, the absence of a uniform definition of AF episodes across the pooled studies, which ranged from 30 s to 3 min, the reliance on study-level data instead of individual participant data, and the inconsistent reporting of anticoagulation uptake after diagnosis.

AF detection after stroke follows a decelerating curve. High-burden episodes are captured within the first days, and the incremental yield of each subsequent monitoring period diminishes as the undetected pool becomes progressively enriched with low-burden, intermittent arrhythmia. This pattern of diminishing returns reflects the depletion of the most readily detected cases. Attrition acts in the same direction. Patients in whom AF is identified early, or who sustain a recurrent event, leave the monitored population, so that those remaining under observation at later time points are fewer and selected toward a lower arrhythmic burden. Whether the observed time-related deceleration reflects a genuine concentration of detectable AF in the acute phase is therefore unknown.

Both studies suggest that earlier monitoring increases and accelerates AF detection, but neither was intended to determine whether earlier detection reduces recurrent stroke by earlier anticoagulation initiation, and the authors explicitly identify this as a question for future research.

## Timing of anticoagulation after AF detection

The safe interval for initiating anticoagulation after AF-associated ischemic stroke has been substantially clarified.^16^ Randomized trials, including ELAN^17,18^ (Early versus Later Anticoagulation for Stroke with Atrial Fibrillation), TIMING^13^ (Early Versus Delayed Non–Vitamin K Antagonist Oral Anticoagulant Therapy After Acute Ischemic Stroke in Atrial Fibrillation), START^19^ (Optimal Delay Time to Initiate Anticoagulation After Ischemic Stroke in Atrial Fibrillation), and OPTIMAS^20^ (Optimal Timing of Anticoagulation After Acute Ischemic Stroke With Atrial Fibrillation), and the CATALYST^21^ (Collaboration on the Optimal Timing of Anticoagulation After Ischaemic Stroke and Atrial Fibrillation) individual-participant-data meta-analysis of these trials, have demonstrated that early initiation of a direct oral anticoagulant (DOAC) is non-inferior to delayed initiation and is associated with low rates of symptomatic intracranial hemorrhage. In CATALYST, initiation within 4 days was associated with a lower 30-day incidence of the composite of recurrent ischemic stroke, symptomatic intracranial hemorrhage, or unclassified stroke than later initiation (2.12% vs. 3.02%; OR = 0.70, 95% CI = 0.50–0.98), with comparably low hemorrhage rates in both groups.

Nash et al.^22^ extend OPTIMAS with a prespecified analysis stratified by infarct volume and location, quantified by central, segmentation-based volumetry in 3572 participants. The effect of early versus delayed initiation did not differ across infarct volumes, with no significant interaction between treatment timing and infarct volume. The incidence of symptomatic intracranial hemorrhage was not increased by early initiation in large infarcts exceeding 25 mL (3/238, 1.3% vs. 5/239, 2.1%). These findings indicate that, within the range of infarct volumes well represented in OPTIMAS, infarct size alone did not identify patients in whom anticoagulation should be delayed; evidence remains less precise for very large infarcts, and they are concordant with the infarct-size-stratified results of ELAN. It must be noted that very large infarcts were uncommon in OPTIMAS only 187/3572 (5.2%) had infarcts > 50 mL and 46/3572 (1.3%) had infarcts > 100 mL. So, the conclusion should not sound definitive for very large infarcts.

Importantly, the population of OPTIMAS and most clinical trials of early versus late anticoagulation comprised patients with known AF or ECG-confirmed AF. Therefore, the results cannot be extrapolated to AFDAS. The available evidence therefore applies most directly to high-burden AF with an established indication for anticoagulation. Whether AFDAS patients derive net benefit from anticoagulation and the optimal timing of its initiation remains undetermined.

## Increased AF detection recurrent stroke risk

Continuous cardiac monitoring increases AF detection and anticoagulation initiation, but whether this strategy reduces recurrent ischemic stroke remains unproven.^6^

Two complementary lines of evidence indicate that this discrepancy is biologically coherent. First, the randomized trials of anticoagulation in ESUS, including NAVIGATE-ESUS^23^ (New Approach Rivaroxaban Inhibition of Factor Xa in a Global Trial Versus ASA to Prevent Embolism in Embolic Stroke of Undetermined Source), RE-SPECT ESUS^24^ (Randomized, Double-Blind, Evaluation in Secondary Stroke Prevention Comparing the Efficacy and Safety of the Oral Thrombin Inhibitor Dabigatran Etexilate versus Acetylsalicylic Acid in Patients with Embolic Stroke of Undetermined Source), ATTICUS^25^ (Apixaban for the Treatment of Embolic Stroke of Undetermined Source), and ARCADIA^26^ (Apixaban to Prevent Recurrence After Cryptogenic Stroke in Patients With Atrial Cardiopathy), were neutral, which argues against the premise that occult AF or atrial cardiopathy accounts for most recurrences in this population.^3^ Second, in patients without a recent stroke, the trials of anticoagulation for device-detected subclinical AF diverged: NOAH-AFNET 6^27^,^28^ (Non–Vitamin K Antagonist Oral Anticoagulants in Patients With Atrial High-Rate Episodes) was stopped early for futility, whereas ARTESiA^29,30^ (Apixaban for the Reduction of Thrombo-Embolism in Patients With Device-Detected Sub-Clinical Atrial Fibrillation) showed a significant reduction in ischemic stroke that was offset by an increase in major bleeding and occurred at low absolute event rates. Furthermore, the populations of the latter studies showed considerable discrepancies compared to those of patients with acute ischemic stroke.^31^ These findings indicate that the low-burden AF that prolonged monitoring increasingly detects carries a lower embolic risk than the persistent, high-burden AF for which anticoagulation was established.

The inference that detecting more AF after stroke will prevent more recurrent strokes is therefore biologically plausible but untested. Resolving it requires trials designed with recurrent stroke as the primary endpoint, such as FIND-AF2^32^ (Intensive Heart Rhythm Monitoring to Decrease Ischemic Stroke and Systemic Embolism: The Find-AF 2 Study), which randomizes patients to intensive versus standard cardiac monitoring.

## AF detection after transient ischemic attack

Most evidence on post-stroke monitoring derives from ischemic stroke, whereas TIA has been comparatively understudied. Veltkamp et al.^33^ address this gap with an updated systematic review and meta-analysis restricted to TIA, comprising 42 studies and 3981 patients. The pooled AF detection rate was 6.5% and increased with monitoring duration, from 3.5% at day 1 to 6.3% at 7 days, 9.6% at 30 days, 13.1% at 90 days, and 19.1% at 12 months. ICM detected substantially more AF than non-invasive monitoring (20.8% vs. 4.7%). The principal contribution is the demonstration that detection rates after TIA approach those reported after ischemic stroke, leading the authors to conclude that newly detected AF is more frequent after TIA than previously recognized. This conclusion is supported by independent data from POINT^34^ (Platelet-Oriented Inhibition in New TIA and Minor Ischemic Stroke), in which the 90-day risk of a new AF diagnosis was similar after TIA and minor ischemic stroke (2.0% vs. 2.7% by the original definition, and 1.8% vs. 2.7% after reclassification by neuroimaging). The index event type had negligible predictive value. These findings support the application of similar rhythm-monitoring strategies to patients with TIA and patients with ischemic stroke, if the main goal is AF diagnosis. However, the comparative ischemic stroke risk of newly diagnosed AF in patients with TIA and ischemic stroke is unknown.

Veltkamp et al.^33^ observed that AF detection in selected TIA cohorts, defined by older age, an undetermined cause, and more extensive cardiovascular evaluation, was approximately twice that in unselected cohorts (9% vs. 4.5%). They demonstrate, however, that this difference is confounded by monitoring modality, because all patients who underwent ICM were preselected, and no difference between selected and unselected cohorts persisted among those undergoing non-invasive monitoring. The authors further note that the absence or small size of infarcts in most patients with TIA renders a neurogenic contribution to AF less likely than after ischemic stroke, such that the mechanistic composition of AF detected after TIA may differ from that after major stroke. Inconsistent use of brain imaging, which permits the inclusion of TIA mimics, incomplete reporting of risk scores such as ABCD2, and substantial between-study heterogeneity further constrain the pooled estimates, which the authors appropriately characterize as exploratory.

The evidence gaps are wider in this population than among ischemic stroke patients (Table 1). As the authors highlight, no randomized trial has examined whether AF detection during prolonged monitoring improves clinical outcomes specifically after TIA. The benefit of anticoagulation for the brief, low-burden episodes typically identified is uncertain, and validated thresholds for AF burden, as well as validated TIA-specific risk scores, are lacking. Research priorities therefore include dedicated trials in patients with TIA.

**Table 1.** Knowledge gaps in AFDAS.

Domain	Knowledge gap
Mechanism	The contribution of neurogenic mechanisms to AFDAS is unknown; predictor associations do not establish causation, and the strongest associations are cardiogenic or stroke severity markers.
Timing and yield of monitoring	Whether the higher and faster detection achieved by earlier monitoring reflects genuine acute-phase arrhythmia or is partly attributable to attrition and diminishing returns is unknown.
Detection and clinical outcomes	It is unproven that detecting more AF, or detecting it earlier, reduces recurrent ischemic stroke. No randomized trial or meta-analysis of prolonged monitoring has shown an outcome benefit.
Anticoagulation in AFDAS	Whether patients with low-burden AFDAS benefit from anticoagulation and the optimal timing of initiation is undetermined. The timing trials enrolled patients with high-burden or ECG-confirmed AF and do not extrapolate. No validated AF burden thresholds exist to guide treatment.
AF after transient ischemic attack	The comparative recurrent stroke risk of newly diagnosed AF after TIA versus ischemic stroke is unknown. No randomized trial has tested whether monitoring improves outcomes after TIA. TIA-specific risk scores are unavailable.

## Conclusion

The five studies in this issue advance the characterization, detection, and treatment of AFDAS, yet they also delineate the questions that remain unresolved. AFDAS is a mechanistically heterogeneous entity whose incidence depends on the detection method, and AF identified on a standard ECG should be distinguished from AF detected by prolonged monitoring. Earlier and more intensive monitoring increases and accelerates detection, although the incremental yield diminishes over time and is shaped by depletion of the most readily detected cases and by attrition. Once AF is identified, early anticoagulation is safe across infarct sizes, but this evidence derives from patients with high-burden, ECG-documented AF and cannot be extrapolated to true AFDAS (detected on continuous cardiac monitoring). The assumption that increased detection translates into fewer recurrent strokes remains untested, and resolving it should be the priority for future research.

## References

[1] Sposato LA Field TS Schnabel RB , et al. Towards a new classification of atrial fibrillation detected after a stroke or a transient ischaemic attack. Lancet Neurol 2024; 23: 110–122. 37839436 10.1016/S1474-4422(23)00326-5

[2] Sposato LA Chaturvedi S Hsieh CY Morillo CA Kamel H. Atrial fibrillation detected after stroke and transient ischemic attack: a novel clinical concept challenging current views. Stroke 2022; 53: e94–e103. 10.1161/STROKEAHA.121.034777 34986652

[3] Sposato LA Sur NB Katan M , et al. Embolic stroke of undetermined source: new data and new controversies on cardiac monitoring and anticoagulation. Neurology 2024; 103: e209535. 10.1212/WNL.0000000000209535 38861698

[4] Sposato LA Cipriano LE Saposnik G Ruíz Vargas E Riccio PM Hachinski V. Diagnosis of atrial fibrillation after stroke and transient ischaemic attack: a systematic review and meta-analysis. Lancet Neurol 2015; 14: 377–387. 25748102 10.1016/S1474-4422(15)70027-X

[5] Cerasuolo JO Cipriano LE Sposato LA. The complexity of atrial fibrillation newly diagnosed after ischemic stroke and TIA: advances and uncertainties. Curr Opin Neurol 2017; 30: 28–37. 27984303 10.1097/WCO.0000000000000410 PMC5321114

[6] Romoli M Urbinati G Tudisco V , et al. Risk of recurrent stroke, mortality, and intracerebral hemorrhage in patients with atrial fibrillation detected before or after a stroke. Neurology 2025; 104: e213426. 10.1212/WNL.0000000000213426 39999395

[7] Alvarado-Bolaños A Ayan D Khaw AV , et al. Differences in stroke recurrence risk between atrial fibrillation detected on ECG and 14-day cardiac monitoring. Stroke 2023; 54: 2022–2030. 37377007 10.1161/STROKEAHA.123.043672

[8] Alvarado-Bolanos A Ayan D Lodol F , et al. Time-varying differences in stroke recurrence risk between types of atrial fibrillation based on screening methods and timing of detection. Eur Stroke J 2025; 10: 461–468. 39589013 10.1177/23969873241300888 PMC11600412

[9] Ababneh GE Yassin A Allahham M , et al. Prevalence and predictors of atrial fibrillation detected after stroke or transient ischemic attack: a comprehensive meta-analysis. Int J Stroke 2025; 21: 910 922. 10.1177/17474930251398615 41201080

[10] Healey JS Connolly SJ Gold MR , et al. Subclinical atrial fibrillation and the risk of stroke. N Engl J Med 2012; 366: 120–129. 22236222 10.1056/NEJMoa1105575

[11] Brambatti M Connolly SJ Gold MR , et al. Temporal relationship between subclinical atrial fibrillation and embolic events. Circulation 2014; 129: 2094–2099. 24633881 10.1161/CIRCULATIONAHA.113.007825

[12] Martin DT Bersohn MM Waldo AL , et al. Randomized trial of atrial arrhythmia monitoring to guide anticoagulation in patients with implanted defibrillator and cardiac resynchronization devices. Eur Heart J 2015; 36: 1660–1668. 25908774 10.1093/eurheartj/ehv115

[13] Doehner W Boriani G Potpara T , et al. Atrial fibrillation burden in clinical practice, research, and technology development: a clinical consensus statement of the European Society of Cardiology Council on Stroke and the European Heart Rhythm Association. Europace 2025; 27: euaf019. 10.1093/europace/euaf019 PMC11901050 40073206

[14] D’Anna L Maguidhir F Simister R , et al. Timing of insertable cardiac monitor implantation after embolic stroke of undetermined source and its impact on atrial fibrillation detection: a target trial emulation analysis. Int J Stroke 2026; 21: 968 978. 10.1177/17474930261438742 PMC13392176 41877402

[15] D’Anna L Prandin G Foschi M , et al. Early versus delayed insertable cardiac monitor implantation after ESUS stroke and the yield of atrial fibrillation detection: a systematic review and meta-analysis. Int J Stroke 2025; 21: 923 932. 10.1177/17474930251404336 PMC13392177 41294253

[16] Johansen MC Sposato LA. Advances in neurocardiology: timing of anticoagulation in patients with ischemic stroke and atrial fibrillation. Stroke 2025; 56: 3306–3309. 41144584 10.1161/STROKEAHA.125.049901

[17] Fischer U Koga M Strbian D , et al. Early versus later anticoagulation for stroke with atrial fibrillation. N Engl J Med 2023; 388: 2411–2421. 37222476 10.1056/NEJMoa2303048

[18] Oldgren J Åsberg S Hijazi Z , et al. Early versus delayed non-vitamin K antagonist oral anticoagulant therapy after acute ischemic stroke in atrial fibrillation (TIMING): a registry-based randomized controlled noninferiority study. Circulation 2022; 146: 1056–1066. 36065821 10.1161/CIRCULATIONAHA.122.060666 PMC9648987

[19] Warach SJ Davis LA Lawrence P , et al. Optimal delay time to initiate anticoagulation after ischemic stroke in atrial fibrillation: a pragmatic, response-adaptive randomized clinical trial. JAMA Neurol 2025; 82: 470–476. 40163159 10.1001/jamaneurol.2025.0285 PMC11959473

[20] Werring DJ Dehbi HM Ahmed N , et al. Optimal timing of anticoagulation after acute ischaemic stroke with atrial fibrillation (OPTIMAS): a multicentre, blinded-endpoint, phase 4, randomised controlled trial. Lancet 2024; 404: 1731–1741. 10.1016/S0140-6736(24)02197-4 39491870

[21] Dehbi HM Fischer U Åsberg S , et al. Collaboration on the optimal timing of anticoagulation after ischaemic stroke and atrial fibrillation: a systematic review and prospective individual participant data meta-analysis of randomised controlled trials (CATALYST). Lancet 2025; 406: 43–51. 40570866 10.1016/S0140-6736(25)00439-8

[22] Nash PS Best JG Lyon J , et al. Early versus delayed anticoagulation in acute ischemic stroke with atrial fibrillation according to infarct volume and location: a prespecified subgroup analysis of the OPTIMAS randomized controlled trial. Int J Stroke 2026; 21: 956 967. 10.1177/17474930261441297 PMC13392158 41906919

[23] Hart RG Sharma M Mundl H , et al. Rivaroxaban for stroke prevention after embolic stroke of undetermined source. N Engl J Med 2018; 378: 2191–2201. 29766772 10.1056/NEJMoa1802686

[24] Diener HC Sacco RL Easton JD , et al. Dabigatran for prevention of stroke after embolic stroke of undetermined source. N Engl J Med 2019; 380: 1906–1917. 31091372 10.1056/NEJMoa1813959

[25] Poli S Meisner C Poli K , et al. Apixaban for treatment of embolic stroke of undetermined source–ATTICUS randomized trial. Eur Stroke J 2022; 7: 24. 10.1177/1747493016681019 27881833

[26] Kamel H Longstreth WT Jr. Tirschwell DL , et al. Apixaban to prevent recurrence after cryptogenic stroke in patients with atrial cardiopathy: the ARCADIA randomized clinical trial. JAMA 2024; 331: 573–581. 38324415 10.1001/jama.2023.27188 PMC10851142

[27] Kirchhof P Toennis T Goette A , et al. Anticoagulation with edoxaban in patients with atrial high-rate episodes. N Engl J Med 2023; 389: 1167–1179. 37622677 10.1056/NEJMoa2303062

[28] Diener HC Becher N Sehner S , et al. Anticoagulation in patients with device-detected atrial fibrillation with and without a prior stroke or transient ischemic attack. The NOAH-AFNET 6 trial. J Am Heart Assoc 2024; 13: e036429. 10.1161/JAHA.124.036429 PMC11646511 39190564

[29] Healey JS Lopes RD Granger CB , et al. Apixaban for stroke prevention in subclinical atrial fibrillation. N Engl J Med 2024; 390: 107–117. 37952132 10.1056/NEJMoa2310234

[30] Shoamanesh A Field TS Coutts SB , et al. Apixaban versus aspirin for stroke prevention in people with subclinical atrial fibrillation and a history of stroke or transient ischaemic attack: subgroup analysis of the ARTESiA randomised controlled trial. Lancet Neurol 2025; 24: 140–151. 39862882 10.1016/S1474-4422(24)00475-7

[31] Sposato LA Wachter R. Anticoagulation in patients with device-detected atrial fibrillation and prior stroke. Lancet Neurol 2025; 24: 92–94. 39862889 10.1016/S1474-4422(24)00524-6

[32] Uhe T Wasser K Weber-Krüger M , et al. Intensive heart rhythm monitoring to decrease ischemic stroke and systemic embolism-the Find-AF 2 study-rationale and design. Am Heart J 2023; 265: 66–76. 37422010 10.1016/j.ahj.2023.06.016

[33] Veltkamp AW Korompoki E D’Anna L , et al. Cardiac monitoring for detection of atrial fibrillation after TIA: an updated systematic review and meta-analysis. Int J Stroke 2026; 21: 933 945. 10.1177/17474930261449734 42056873

[34] Kamel H Farrant M Easton JD , et al. Newly diagnosed atrial fibrillation after transient ischemic attack versus minor ischemic stroke in the POINT Trial. J Am Heart Assoc 2021; 10: e019362. 10.1161/JAHA.120.019362 PMC8174230 33682440

